# Preventing and reducing violence against women: innovation in community-level studies

**DOI:** 10.1186/s12916-014-0155-9

**Published:** 2014-10-01

**Authors:** Angela Taft, Rhonda Small

**Affiliations:** Judith Lumley Centre, La Trobe University, Melbourne, Australia

**Keywords:** Intimate partner violence, Community randomized controlled trial, Prevention, Intervention

## Abstract

Intimate partner violence is a serious global problem that damages the health and prosperity of individuals, their families, community, and society. WHO endorses an ‘ecological model,’ which states that there are multi-level intersecting factors enabling perpetration and victimization of violence. Intervention science to prevent or reduce the problem is in its infancy, and the few existing intervention studies have been targeted at the individual level. In a recent study published in *BMC Medicine*, Abramsky *et al*. bring innovation to the field, targeting their intervention trial “SASA!” in Kampala Uganda at all ecological levels, but particularly at the community level. Recruiting and training both male and female community leaders and activists who enabled group and media discussions, the authors focused on the beneficial and abusive detrimental uses of power rather than commencing with the central issue of gender inequality. SASA! successfully reduced community attitudes to tolerance of violence and inequality, men’s sexual risk behaviors, and women’s experience of physical violence. The study also improved the communities’ response to victimized women. SASA! has promise for adaptation and replication in low, middle and high income countries.

Please see related article: http://www.biomedcentral.com/1741-7015/12/122.

## Background

Gender-based violence, especially its most common form – intimate partner violence (IPV) – is prevalent globally. Evidence that such violence causes serious health damage to women, their children, families, and society is now overwhelming [1]. It can be particularly harmful to the health and development of low and middle income countries (LMIC), for example, affecting maternal morbidity and mortality rates, and the levels of HIV infection among women and children. In an article recently published in *BMC Medicine*, Abramsky *et al.* demonstrated innovation in methods for pragmatic randomized trials for the prevention of gender-based violence [2]. In doing so, they advanced the very small evidence base of effective interventions to prevent and reduce the level of IPV and sexual violence against women and the consequences of such violence.

**Figure 1:**
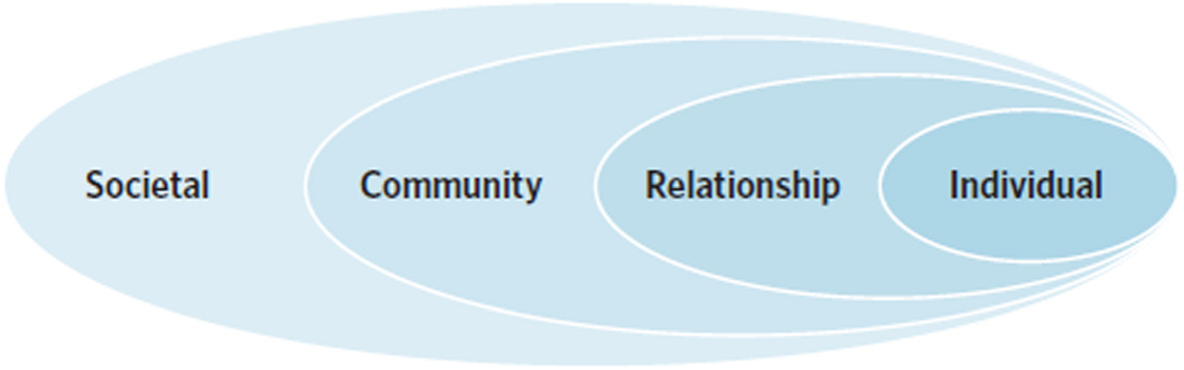
**The ecological model.** Reproduced from [3] with permission from the World Health Organisation.

The World Health Organization (WHO) recently published a strategic framework for preventing and reducing violence against women [3]. In doing so, WHO drew on an ‘ecological model’ of factors (below, Figure 1), which illustrates the intersecting determinants of IPV that can influence the likelihood that men will abuse women and women will become victimized.

The factors related to both victimization and perpetration, and therefore to finding solutions to IPV, are located at the level of the individual (for example, witnessing abuse as a child; drinking alcohol at harmful levels), the relationship (for example, men controlling financial resources or having multiple partners); the community (high poverty or unemployment levels, weak community sanctions) and society (for example, norms of masculinity including dominance and aggression; the absence of legal sanction or redress against gender-based violence) [3].

In the SASA! Study, Abramsky *et al.* identified gender inequality and the consequent power imbalance between women and men as central to an environment in which violence against women can flourish [2]. They understood the need for the development and rigorous evaluation of interventions to prevent and reduce IPV and sexual violence against women at all levels of the WHO model, and decided to target their intervention predominantly at the community level. The authors of the WHO Prevention report reviewed the current evidence for effective interventions to prevent and reduce partner violence, and found it to be very inadequate. The authors (p.1) concluded that ‘The primary prevention of these types of violence will … save lives and money – investments made now to stop IPV and sexual violence before they occur will protect the physical, mental and economic well-being and development of individuals, families, communities and whole societies’ [3].

There have been several systematic reviews of partner violence interventions, predominantly targeted at individuals, for example, using advocacy [4] or using clinician practices such as screening for partner violence in healthcare settings [5]. Such reviews have also found the evidence base to be small. In addition, the majority of the evidence comes from high income countries (HIC), notably the USA, and may therefore not be applicable in resource-poor countries. From existing studies, there is some evidence that advocacy (providing identified individual women victims/survivors with information and support) can improve women’s health and well-being; that psychological interventions (for example, psychotherapeutic methods) can improve pregnant women’s outcomes [6]; and that interventions by primary care clinician may increase identification, referrals, and depression [7,8].

Rigorously developed and evaluated interventions on IPV in LMIC are very few, but those that exist have taken a broader perspective focused on known determinants of partner abuse or on implemented and innovative approaches to social support. These have targeted individual women, for example, initiatives aimed at building alliances between mothers-in-law and daughters-in-law in India to stop abuse of pregnant and post-partum women [9]; micro-financing and gender and advocacy training [10]; and tackling gender norms and economic empowerment [11].

The innovation that SASA! [2] (Start, Awareness, Support, Action!), a cluster randomized trial conducted in Kampala, Uganda has successfully demonstrated is that it is possible to change gender norms and attitudes by targeting interventions to reduce partner violence and HIV behaviors at the community level. SASA! used the ecological model above as an intervention framework (targeting societal, community, relationship, and individual attitudes and practices). It aimed to engage not only women but also men (involving many degrees of difficulty in attempting to change male and female attitudes and behaviors). Working to identify community leader/partners and to build alliances is a marathon effort, and the fruits of these efforts can also take time to appear. There were other challenges; for example, during the trial implementation, political conflict and elections in Uganda interrupted the activities, and the study had to be suspended for a time.

The major strategies in SASA! involved training male and female community leaders and community activists in the four intervention communities, after which the participants engaged in critical discourse in the community media and with community groups about power and power inequalities. Their message was not only how power can be abused. but also how it can be used positively for beneficial change. The pre-defined primary outcomes included: 1) reduced social acceptance of gender inequality and IPV; 2) decrease in experience of IPV; 3) improved (community) response to women experiencing violence; and 4) decrease in sexual risk behaviors. High rates of activity, participation, and improved community response to violence led to significant reductions in the acceptance of men’s use of violence and increased acceptance of a woman’s right to refuse sex in intervention communities. Men’s concurrent sexual partners (a risk factor for HIV infection) and women’s experience of physical violence in the past year were also reduced.

The SASA! study authors argue that these effects at community level, not limited to people with high levels of exposure, attest to the diffusion that can occur in communities and the importance of community-level studies. An improved response to victimized women at community level consolidates this. The study acknowledged some limitations (potential contamination that might have weakened effects, and issues of population mobility); nevertheless this should not diminish the importance of the outcomes achieved.

## Conclusions

This trial will hopefully stimulate further community-level intervention studies in other LMIC, and indeed also in HIC, where individual approaches have been more common and have proved inadequate to date. Importantly for future research, the involvement in SASA! of male as well as female advocates to challenge attitudes to power and partner violence, proved both innovative and effective in reducing violence. Reducing IPV and other forms of gender-based violence will improve the health, social welfare, and economy, particularly of LMIC.
